# The role of heparin in preventing early thrombosis of arteriovenous fistula for hemodialysis: An observational Study

**DOI:** 10.6026/973206300211149

**Published:** 2025-05-31

**Authors:** Aandrei Jivendra Jha, Tushar Kumar, Madhav Kumar, Alok Bharti, Ruchi Singh

**Affiliations:** 1 Department of Cardiothoracic and Vascular Surgery, Indira Gandhi Institute of Medical Sciences, Sheikhpura, Patna, Bihar, India; 2 Department of Anesthesia, Indira Gandhi Institute of Medical Sciences, Sheikhpura, Patna, Bihar, India; 3 Department of Cardiothoracic and Vascular Surgery, Patna Medical College - PMC, Ashok Rajpath Rd, Patna University Campus, Patna, Bihar, India

**Keywords:** Arteriovenous fistula, hemodialysis, early thrombosis, heparin, vascular access, anticoagulation, fistula maturation, dialysis access patency

## Abstract

Arteriovenous fistula (AVF) is preferred for hemodialysis. However, early thrombosis often leads to failure. Hence, this observational study of 200 patients compared AVF outcomes with and without intraoperative heparin use. Thrombosis within 30 days was significantly lower in the heparin group (11% vs. 25%, p = 0.01). Smaller vein diameter was an independent risk factor, while heparin improved maturation and short-term patency. Despite minor bleeding, heparin prophylaxis was safe and may reduce early AVF failure.

## Background:

For vascular access in haemodialysis patients, the arteriovenous fistula (AVF) is the gold standard due to its superior long-term patency and lower risk of complications compared to central venous catheters and grafts [1]. However, because early thrombosis often leads to repeated treatments, delayed dialysis initiation and access failure, it remains a significant concern [2]. Heparin is an anticoagulant commonly used in vascular procedures that is believed to reduce the risk of thrombosis by lowering clot formation in the critical early post-operative period [3]. There may be benefits to heparin prophylaxis, although the optimal dosage and bleeding concerns remain controversial. Multiple investigations have suggested that decreased preoperative vein diameter, systemic comorbidities and perioperative haemodynamic may influence AVF maturation and patency [4]. This study aims to evaluate the effects of heparin on maturation rates and its ability to prevent early AVF thrombosis in haemodialysis patients at IGIMS. The trade-off between thrombotic risk reduction and bleeding issues must be understood in order to maximise AVF results and prolong the life of vascular access in dialysis-dependent patients [5, 6]. Therefore, it is of interest to report the role of heparin in preventing early thrombosis of arteriovenous fistula for hemodialysis.

## Methodology:

This observational study was conducted at IGIMS, enrolling 200 hemodialysis patients who underwent AVF creation to assess the intraoperative heparin's role in the prevention of early thrombosis. The patients were divided into two groups: patients receiving systemic heparin prophylaxis and those who were not receiving it and rates of thrombosis between them were compared over the first 30 days of surgery. Patient demographics, preoperative diameter of the vein, comorbidities, use of anticoagulation and AVF outcome were gathered. The primary result was premature AVF thrombosis, while secondary results were fistula maturation rates, patency and bleeding complications. Statistical comparison with the appropriate tests was done, using multivariate regression to adjust for confounding variables of AVF success. Results aim to give clinical insight into the safety and efficacy of heparin in AVF management that can be used in future anticoagulation protocols for hemodialysis patients.

## Results:

Depending on how much heparin they used during the surgery, 200 haemodialysis patients who had arteriovenous fistulas (AVFs) made were divided into two groups. The incidence of early AVF thrombosis within 30 days was significantly lower in the heparin group (11%) compared to the non-heparin group (25%; p = 0.01). A strong predictor of thrombosis was preoperative vein width and smaller veins were associated with a higher likelihood of early failure. Although not statistically significant, there was a tendency towards radiocephalic fistulas in the location of AVFs (radiocephalic vs. brachiocephalic; 15% vs. 23% thrombosis rate) (p = 0.12). There was no significant correlation between the risk of thrombosis and comorbid diseases such as diabetes or hypertension. Although the heparin group experienced mild bleeding issues, there were no notable hemorrhagic incidents. The study findings show that preoperative vein diameter was significantly associated with early AVF thrombosis, with smaller veins increasing the risk of failure (Table 1). Intraoperative heparin use significantly reduced thrombosis rates, with 11% incidence in the heparin group compared to 25% in the non-heparin group (p = 0.01), confirming its protective effect (Table 2). Although radiocephalic AVFs showed a lower thrombosis rate than brachiocephalic AVFs, this difference was not statistically significant (Table 3). Diabetes and hypertension did not demonstrate a significant association with thrombosis risk (Table 4), suggesting that systemic conditions alone may not be strong predictors of AVF failure. While minor bleeding was slightly higher in the heparin group (p = 0.03), no major hemorrhagic events occurred, reinforcing the overall safety of heparin administration (Table 5). While closely monitoring minor bleeding problems, these findings support the routine use of intraoperative heparin to improve AVF patency and lower the risk of early thrombosis in haemodialysis patients.

## Discussion:

The implications of the research point towards the important function of intraoperative heparin in lowering rates of early thrombosis of arteriovenous fistulas (AVFs) in hemodialysis patients [7, 8]. Heparin patients demonstrated a dramatically reduced rate of thrombosis (11%) versus non-heparin patients (25%) and its effectiveness at preserving vascular patency within the early post-surgical phase was indicated [9, 10]. Preoperative vein diameter was recognized as a significant predictor of thrombosis, with lower veins at higher risk of early failure, highlighting the value of thorough preoperative evaluation [11]. Although radiocephalic AVFs had lower rates of thrombosis than brachiocephalic AVFs, the result was not statistically significant; suggesting that location per se may not be the only determining factor for fistula patency [12]. Diabetes and hypertension comorbid conditions did not present a significant relationship with early thrombosis, indicating that it is not just systemic disease predicting early AVF failure [13]. Minor hemorrhagic complications occurred more often in the heparin arm but no major hemorraghic complications were noticed, attesting to the global safety of anticoagulation in this indication [14]. The findings concur with the current literature that supports the use of anticoagulation in high-risk AVFs but also indicate the necessity of achieving an optimal balance between preventing thromboses and avoiding bleeding risk. Standardized heparin dosing protocols need to be studied in the future to reduce complications while providing maximal benefit in protection of AVFs [15]. Regular intraoperative heparin usage may enhance long-term dialysis access results and ultimately decrease the need for repeated interventions, as early thrombosis continues to be a major cause of access failure. In order to improve fistula survival in haemodialysis patients, these results highlight the significance of early anticoagulation techniques and customised vascular access planning.

## Conclusion:

Intraoperative heparin use significantly reduced early thrombosis rates in arteriovenous fistulas (AVFs) compared to non-heparin controls, with no major bleeding complications reported. While minor bleeding was more frequent, the benefits in maintaining vascular patency outweighed the risks. These findings support routine heparin use during AVF creation, though further research is needed to standardize dosing protocols for optimal safety and efficacy.

## Figures and Tables

**Table 1:** Demographics and clinical characteristics

**Variable**	**Total (n = 200)**	**Thrombosis (n = 36)**	**No Thrombosis (n = 164)**	**p-value**
Age (years)	52.3 ± 12.7	54.1 ± 11.9	51.8 ± 12.8	0.25
Male (%)	58	61	57	0.68
Diabetes (%)	45	50	44	0.54
Hypertension (%)	62	67	60	0.48
Preoperative Vein Diameter (mm)	2.4 ± 0.4	2.1 ± 0.3	2.5 ± 0.4	0.02

**Table 2:** Association of intraoperative heparin with early AVF thrombosis

**Heparin Use**	**Total (n = 200)**	**Thrombosis (n = 36)**	**No Thrombosis (n = 164)**	**p-value**
Administered (%)	50	11	89	0.01
Not Administered (%)	50	25	75	

**Table 3:** Influence of AVF location on thrombosis

**AVF Location**	**Total (n = 200)**	**Thrombosis (n = 36)**	**No Thrombosis (n = 164)**	**p-value**
Radiocephalic (%)	70	15	85	0.12
Brachiocephalic (%)	30	23	77	

**Table 4:** Impact of comorbidities on thrombosis-free patency

**Comorbidity**	**Total (n = 200)**	**Thrombosis (n = 36)**	**No Thrombosis (n = 164)**	**p-value**
Diabetes (%)	45	50	44	0.54
Hypertension (%)	62	67	60	0.48

**Table 5:** Complications related to heparin

**Complication**	**Heparin Group (%)**	**Non-Heparin Group (%)**	**p-value**
Minor Bleeding	12	5	0.03
Major Bleeding	0	0	-
